# Correlation of sociodemographic profiles with psychological problems among hospitalized patients receiving unplanned hemodialysis

**DOI:** 10.1080/0886022X.2020.1736097

**Published:** 2020-03-09

**Authors:** Yu-Yin Kao, Wen-Chin Lee, Ruey-Hsia Wang, Jin-Bor Chen

**Affiliations:** a College of Nursing, Kaohsiung Medical University, Kaohsiung, Taiwan; b Department of Nursing, Kaohsiung Chang Gung Memorial Hospital, Kaohsiung, Taiwan; c Division of Nephrology, Department of Internal Medicine, Kaohsiung Chang Gung Memorial Hospital, Kaohsiung, Taiwan; d Chang Gung University College of Medicine, Taoyuan, Taiwan; e College of Nursing, Department of Medical Research, Kaohsiung Medical University, Kaohsiung Medical University Hospital, Kaohsiung, Taiwan

**Keywords:** unplanned hemodialysis, anxiety, depression, sleep disturbance

## Abstract

**Purpose:**

In this prospective study, we aimed to examine the sociodemographic factors and clinical factors associated with psychological disorders in chronic kidney disease (CKD) patients receiving unplanned hemodialysis (HD).

**Methods:**

We prospectively enrolled 187 CKD stage 5 patients receiving unplanned HD at a tertiary hospital from January 2015 to December 2016. We used structured questionnaires to gather data about participants’ anxiety, depression, and sleep disturbance. Generalized linear regression analysis was used to examine the relationships between sociodemographic and laboratory parameters, and severity of psychological distress.

**Results:**

The mean age of the participants was 60 years, and the number of men and women was 97 and 90, respectively. We did not find a significant association between anxiety, depression, and sleep disturbance scores and gender, age, marital status, religion status, education levels, and employment status and number of comorbidities. Generalized linear regression analysis showed that a multidisciplinary CKD care program in outpatient clinic disclosed a significant negative association with psychological disorders in participants.

**Conclusions:**

CKD patients exhibited psychological distress when receiving unplanned HD, not closely associated with sociodemographic profiles.

## Introduction

The global prevalence of dialysis patients has increased 1.7 times from 165 per million population (pmp) in 1990 to 284 pmp in 2010 [1]. In Taiwan, the incidence of dialysis patients was 492.4 pmp and the prevalence of dialysis patients was 3177.8 pmp according to the 2016 annual report [2]. Accumulating evidence has shown that patients with chronic kidney disease (CKD) are highly susceptible to emotional problems especially when starting dialysis [3,4]. Similar to many chronic illnesses, CKD status delivers disease burden, functional limitation, dietary restriction, adverse effects of medication, modified social behavior, and fear of dialysis therapy. A longitudinal psycho-social impact influences quality of life causing more functional impairment, greater psychological stress and lower adherence to drug treatment in CKD patients [5–8]. Consequently, there is increased morbidity and risk of mortality in this population. Therefore, it is important to identify and treat these psychological conditions to improve the overall well-being and quality of life.

Anxiety, depression, and sleep disturbance are the three most common psychiatric/psychological problems in the CKD population. Overall, the prevalence of anxiety, depression, and sleep disturbance were reported to range from 40% to 80% in different CKD populations [5–11]. Anxiety and depression among CKD patients often co-occur and seem to exacerbate each other through combined effects. The causes for sleep disturbance in CKD patients include depression, anxiety, uremic toxins, and adverse effects of drugs, among others. Moreover, previous reports have also indicated that this psychological distress cannot be alleviated when CKD patients begin dialysis therapy [11–16].

Although several studies have been conducted on anxiety, depression, and sleep disturbance in CKD patients, most of them focused on the psychological problems of CKD patients on maintenance dialysis therapy. Until now, there is no report on psychological distress in CKD patients undergoing unplanned hemodialysis (HD).

This prospective study aimed to examine the dynamic changes in this psychological distress and their association with sociodemographic variables in CKD patients receiving unplanned HD. We hope that the findings of this study may provide a reference for appropriate psychological intervention strategies to improve the quality of life of CKD patients.

## Materials and methods

### Study design and patients

Patients who received unplanned HD at Kaohsiung Chang Gung Memorial Hospital in Taiwan were prospectively enrolled for this study. The defined period of recruitment was from January 2015 to December 2016. The inclusion criteria were (1) age ≥20 years; (2) unplanned HD initiation in hospitalized patients; and (3) no renal-replacement therapy prior to recruitment. The exclusion criteria were: (1) having a history of psychiatric illness or taking psychiatric medications before hospitalization and (2) unable to communicate orally. All patients received unplanned HD *via* a temporary tunneled HD catheter either in the femoral vein or internal jugular vein. Indications for unplanned HD included severe acute heart failure (ejection fraction in left ventricle <30%), anasarca with respiratory distress, severe hyperkalemia (serum potassium concentration >6.5 mEq/L), and severe acidosis (serum bicarbonate concentration <10.0 mEq/L). Multidisciplinary CKD care program was integrated in all participants in outpatient clinic. The program included nephrologist, nephrology nurse educator, renal dietitian, social worker, pharmacy specialist and surgeon. Stage 3 or 5 CKD patients were followed up every 3 months, and stage 5 CKD patients were followed up at least every month. In follow-up occasions, participants were instructed for life style modification, dietary content, drug effects and laboratory data explanation. The study was approved by the Kaohsiung Chang Gung Memorial Hospital Institutional Review Board, Taiwan (documentation number: 104-6438c). All participants were interviewed after obtaining written informed consent.

## Data collection

### Laboratory measurement and self-report questionnaires

The self-report questionnaires at baseline were collected by a study nurse two weeks after initial urgent-start HD. Self-report questionnaires assessed participants’ demographic characteristics including age, sex, marital status, educational status, religious status, and employment status. A scoring system was used for evaluation of anxiety status (State–Trait Anxiety Inventory), depression status (Center for Epidemiological Studies Depression, CES-D), and sleep disturbance (Pittsburgh Sleep Quality Index, PSQI). Laboratory-examined data were collected at the time of the first unplanned HD. Medical charts were reviewed to collect information on comorbidities among participants. The collected laboratory variables included hemoglobin (Hb), serum albumin, urea nitrogen, creatinine (Cr), calcium (Ca), inorganic phosphate (P), sodium (Na), potassium (K) and estimated glomerular filtration rate (eGFR). eGFR (mL/min/1.73 m^2^) was calculated based on the Modification of Diet in Renal Disease (MDRD) equation for Chinese individuals: 175 × [serum Cr (mg/dL)] − 1.234 × age −0.179 × 0 79 (if female).

### State-Trait Anxiety Inventory (STAI)

A 20-item Chinese version of the state anxiety scale was used to assess the levels of anxiety [17]. The scale is comprised of 10 negative affect and 10 positive affect items. The total summative score ranges from 20 to 80, where a higher score indicates higher levels of anxiety. Scores of 20–39 indicate mild anxiety, scores between 40 and 59 indicate moderate anxiety, and scores between 60 and 80 indicate severe anxiety [18]. Cronbach’s alpha was 0.98 in this study.

### Center for Epidemiologic Studies Depression Scale (CES-D)

The 20-item Chinese version of Center for Epidemiologic Studies Depression Scale (CES-D) [19], weighted by frequency of occurrence during the past week, was used to assess the depression symptoms. The CES-D is comprised of 16 negative affect and 4 positive affect items. Scores range from 0 to 60, and less than 16 indicates normal; a score between 16 and 20 indicates mild depressive symptoms; a score between 21 and 26 indicates moderate depression symptoms; and a score between 27 and 60 indicates severe depressive symptoms [20]. In this study, Cronbach’s alpha was 0.97.

### Pittsburgh Sleep Quality Index (PSQI)

The Chinese version of the Pittsburgh Sleep Quality Index (CPSQI) [21] was used to assess sleep problems over one month. The PSQI is a 19-item self-report questionnaire with seven subscales: subjective sleep quality, sleep latency, sleep duration, habitual sleep efficiency, sleep disturbances, use of sleep medication, and daytime drowsiness. Each item was rated on a four-point scale ranging from 0 to 3, which generates a total score ranging between 0 and 21, with higher scores indicating poorer quality of sleep, while good sleepers had a score of less than 5 [22]. In this study, Cronbach’s alpha was 0.91.

## Data analysis

All data were analyzed using the computer-based statistical software package SPSS, version 18.0. Descriptive statistics were calculated to examine the distribution of the study variables among participants. Categorial variables were used chi-squared or Fisher’s exact test and using an independent two-sample *t*-test for continuous variables. Generalized linear regression analysis was used to examine the associations of measured psychological scores and sociodemographic and laboratory parameters. *p* values of less than 0.05 were considered statistically significant.

## Results

### Baseline characteristics

This study included 187 patients with unplanned HD. Mean age of the study participants at baseline was 60 years, including 90 females and 97 males. Most participants were married (*n* = 120, 64.2%) and had religious beliefs (*n* = 109, 58.3%); however, the unemployment rate reached 66.3%. Moreover, 35.8% participants were educated up to elementary level, while 42.3% patients had secondary education, and only 15% graduated above the college level. The etiologies of CKD among participants were diabetic nephropathy (*n* = 91, 48.7%), renal diseases (*n* = 52, 27.8%) and hypertensive nephrosclerosis (*n* = 25, 13.4%), and undetermined (*n* = 19, 10.2%) (Table 1).

**Table 1.** Baseline characteristics.

Participant characteristics	*n* (%)
Sex	
Male	97 (51.9)
Female	90 (48.1)
Age(years)	59.98 ± 15.19
<65	111 (59.4)
≧65	76 (40.6)
Marital status	
With spouse	120 (64.2)
Single	67 (35.8)
Religion	
No	78 (41.7)
Yes	109 (58.3)
Education	
Illiterate	13 (7.0)
Elementary school	67 (35.8)
Junior high school	31 (16.6)
Senior high school	48 (25.7)
College or above	28 (15.0)
Employment	
No	124 (66.3)
Yes	63 (33.7)
Comorbidity	
0–2	46 (24.6)
3	62 (33.2)
4	62 (33.2)
≧5	17 (9.1)
Diabetes	
No	78 (41.7)
Yes	109 (58.3)
Hypertension	
No	18 (9.6)
Yes	169 (90.4)
Cardiovascular disease	
No	120 (64.2)
Yes	67 (35.8)
Awareness of renal disease	
No	22 (11.8)
Yes	165 (88.2)
Etiology of CKD	
Diabetes	91 (48.7)
Renal disease	52 (27.8)
Hypertension	25 (13.4)
Undetermined	19 (10.2)
Laboratory parameters	
Hb (g/dL)*	7.6 (6.9–8.3)
Albumin (g/dL)*	3.3 (2.9–3.7)
BUN (mg/dL)*	130 (102–160)
Creatinine (mg/dL)*	11.53 (9.40–15.06)
Calcium (mg/dL)*	8.1 (7.4–8.6)
Phosphate (mg/dL)*	6.9 (5.7–8.7)
Sodium (mEq/L)*	135 (132–138)
Potassium (mEq/L)*	4.6 (4.1–5.3)
eGFR (mL/min/1.75 m^2^)*	3.90 (3.00–5.03)

Abbreviations: Hb: hemoglobin; BUN: blood urea nitrogen; eGFR: estimated glomerular filtration rate.

Data were expressed as mean ± SD and *median (interquartile range).

### Stratified severity according to anxiety, depression, and sleep disturbance scores

Out of the 187 participants, more than half (*n* = 110, 58.8%) presented with severe anxiety (anxiety score: 60–80) and severe depression (*n* = 181, 96.8%) (depression score: 27–60). All participants had poor sleep quality (PSQI score ≥5) (Table 2).

**Table 2.** Distribution of scores in anxiety, depression, sleep disturbance in participants.

Variable	*N* (%)	Range	Mean ± SD
Anxiety		25–80	61.90 ± 17.88
Mild	28 (15%)	20–39	32.43 ± 4.13
Moderate	49 (26.2%)	40–59	48.57 ± 5.74
Severe	110 (58.8% )	60–80	75.35 ± 6.46
Depression		23–60	49.03 ± 11.93
Mild	0	16–20	
Moderate	6 (3.2%)	21–26	24.83 ± 1.17
Severe	181 (96.8%)	27–60	49.83 ± 11.26
Sleep disturbance	187	9–21	16.06 ± 4.15

### Comparison of anxiety, depression, and sleep disturbance scores between different sociodemographic profiles

There were no significant differences between anxiety, depression, sleep disturbance, and sociodemographic profile scores including sex, age, marital status, religion status, educational levels, employment status, and awareness of renal disease (Table 3).

**Table 3.** Distribution of scores in anxiety, depression, sleep disturbance based on sociodemographic profile.

Participant characteristics	Anxiety scores	Depression scores	Sleep disturbance scores
Sex			
Male	60.19 ± 17.94	48.10 ± 12.15	15.90 ± 4.15
Female	63.76 ± 17.74	50.13 ± 11.74	16.24 ± 4.16
*t*	−1.367	−1.162	−.571
Age (years)			
<65	62.62 ± 18.65	49.67 ± 12.06	16.32 ± 4.13
≧65	60.86 ± 16.75	48.22 ± 11.86	15.68 ± 4.18
*t*	.662	.809	1.036
Marital status			
Spouse	61.29 ± 17.88	48.57 ± 12.25	15.88 ± 4.04
Single	63.00 ± 17.97	50.00 ± 11.47	16.39 ± 4.36
*t*	−0.625	−0.785	−0.796
Religion			
Yes	61.37 ± 18.54	48.98 ± 11.90	16.14 ± 4.36
No	62.65 ± 17.02	49.22 ± 12.14	15.96 ± 3.87
*t*	−.484	−.133	−.291
Education			
Illiterate	61.85 ± 17.04	48.38 ± 12.32	15.31 ± 4.05
Elementary school	61.54 ± 17.35	48.64 ± 11.94	16.09 ± 4.09
Junior high school	61.16 ± 18.71	48.65 ± 11.84	15.48 ± 4.57
Senior high school	61.46 ± 19.02	49.13 ± 12.79	16.40 ± 3.81
College or above	64.39 ± 17.67	50.86 ± 11.21	16.43 ± 4.60
*F*	.161	.195	.385
Employment			
Yes	63.46 ± 18.06	49.81 ± 12.04	16.37 ± 4.04
No	61.11 ± 17.81	48.71 ± 11.96	15.91 ± 4.22
*t*	.848	.593	.706
Awareness of renal disease			
Yes	60.98 ± 17.80	48.55 ± 12.10	15.90 ± 4.12
No	68.82 ± 17.35	53.05 ± 10.28	17.27 ± 4.28
*T*	1.945	1.662	1.413

Data were expressed as mean ± SD. *p* value were estimated using independent two-sample *t*-test and ANOVA test.

### Distribution of anxiety, depression, and sleep disturbance scores according to number of comorbidities

Most patients (*n* = 141, 75.5%) had at least three comorbidities, including diabetes, hypertension, gout, systemic lupus erythematosus, cerebrovascular disease, or chronic hepatitis. The distribution of three major comorbidities was presented. Hypertension (90.4%) was the leading comorbidity, followed by diabetes (58.3%) and cardiovascular diseases (35.8%). The number of comorbidities in participants did not influence the score distribution for anxiety, depression, and sleep disturbance (Table 4).

**Table 4.** Distribution of scores in anxiety, depression, sleep disturbance based on number of comorbidities.

Participant characteristics	*N* (%)	Anxiety scores	Depression scores	Sleep disturbance scores
Etiology of CKD				
Diabetes	91 (48.7)	62.40 ± 16.00	49.53 ± 11.01	16.22 ± 3.95
Renal disease	52 (27.8)	61.33 ± 18.58	47.85 ± 12.76	15.94 ± 4.35
Hypertension	25 (13.4)	56.40 ± 20.70	45.96 ± 13.43	15.04 ± 4.68
Other	19 (10.2)	68.37 ± 19.70	53.95 ± 10.55	17.00 ± 3.84
* F*		1.676	1.878	.885
Total number of comorbidity				
0–2	46 (24.6)	61.11 ± 19.71	48.15 ± 12.87	15.59 ± 4.52
3	62 (33.2)	63.74 ± 17.69	49.53 ± 12.24	16.16 ± 4.04
4	62 (33.2)	60.94 ± 16.32	49.61 ± 11.17	16.21 ± 3.90
≧5	17 (9.1)	60.88 ± 19.95	48.00 ± 12.09	16.47 ± 4.67
*F*		.324	.206	.290
Diabetes				
No	78 (41.7)	62.19 ± 19.94	48.95 ± 12.88	15.77 ± 4.43
Yes	109 (58.3)	61.70 ± 16.35	49.17 ± 11.33	16.28 ± 3.94
*t*		.186	−.127	−.821
Hypertension				
No	18 (9.6)	72.22 ± 14.83	55.22 ± 9.53	17.83 ± 3.84
Yes	169 (90.4)	60.80 ± 17.87	48.43 ± 12.04	15.88 ± 4.15
*t*		2.615	2.317	1.916
Cardiovascular disease				
No	120 (64.2)	61.23 ± 18.11	48.48 ± 12.23	15.78 ± 4.12
Yes	67 (35.8)	63.10 ± 17.55	50.15 ± 11.50	16.58 ± 4.18
*t*		−.685	−.912	−1.277

Data were expressed as mean ± SD.

*p* value were estimated using independent two-sample *t*-test and ANOVA test.

### Associations of sociodemographic factors and clinical factors with anxiety, depression, and sleep disturbance scores

By generalized linear regression analysis, all measured psychological scores were not associated with examined sociodemographic and laboratory parameters except hypertension showed significant negative association with anxiety scores (adjusted *β* = −0.167, *p* = 0.046). The CKD prevention program also disclosed a significant negative association with anxiety, depression, and sleep disturbance scores (Table 5).

**Table 5.** Results of the generalized linear regression analyses with anxiety, depression and sleep score as outcome variables.

	Anxiety scores		Depression scores		Sleep disturbance scores	
Participant characteristics	Adjusted beta	p Values	Adjusted beta	p Values	Adjusted beta	p Values
Sex	−0.160	0.056	−0.107	0.203	−0.049	0.556
Age	0.071	0.483	0.082	0.422	−0.065	0.520
Marital status	−0.015	0.834	−0.028	0.708	−0.010	0.887
Religion	−0.096	0.184	−0.060	0.406	−0.061	0.393
Education						
<7 years	0.024	0.849	−0.047	0.712	0.066	0.599
>7 years	0.005	0.963	−0.020	0.853	0.051	0.634
Employment	0.041	0.612	0.035	0.666	−0.045	0.569
Comorbidity						
3	0.069	0.453	0.057	0.535	0.065	0.481
4	−0.005	0.961	0.059	0.522	0.071	0.444
≧5	−0.004	0.965	−0.003	0.971	0.061	0.457
Diabetes	0.037	0.767	0.055	0.666	0.210	0.092
Hypertension	−0.167	0.046	−0.134	0.112	−0.100	0.227
Cardiovascular disease	0.052	0.504	0.042	0.591	0.036	0.642
Renal disease	−0.091	0.286	−0.087	0.312	−0.097	0.254
Etiology of CKD						
Diabetes	−0.031	0.853	−0.121	0.473	−0.143	0.389
Renal disease	−0.078	0.517	−0.165	0.178	−0.052	0.664
Hypertension	−0.098	0.365	−0.143	0.191	−0.083	0.438
Laboratory parameters						
Hb (g/dL)	0.081	0.304	0.072	0.362	0.197	0.012
Albumin (g/dL)	−0.092	0.217	−0.041	0.587	−0.111	0.135
eGFR (mL/min/1.73 m^2^)	−0.019	0.879	−0.104	0.414	−0.128	0.308
CKD prevention program	−0.387	0.000	−0.413	0.000	−0.387	0.000
Adjust *R*^2^	0.201		0.185		0.207	

### Correlation analysis among anxiety, depression, and sleep disturbance

Using Pearson’s correlation test, results showed significant correlation between anxiety, depression and sleep disturbance. Detailed information was showed in Supplementary Table S1.

## Discussion

In this study, we studied the psychological problems (i.e., anxiety, depression, and sleep disturbance) in patients with end-stage renal disease who received unplanned HD. We found that the majority of patients presented with severe anxiety, depression, and sleep disturbance when they had to undergo unplanned HD. The overall percentage of these psychological problems was higher in our cohort compared to prior reports of CKD and HD patients [10,11,23–25]. The main reason for the difference is subject selection. Our study only included patients who underwent unplanned HD; however, prior studies included pre-dialysis and planned dialysis patients. Our study also showed that the majority of patients were experiencing severe psychological distress. Accordingly, CKD patients preserved a psychological burden especially when they had to undergo unplanned HD. This scenario strengthened the necessity for timely psychological care in patients at risk for dialysis.

The association between socioeconomic status and psychological disturbance in CKD patients has been assessed in previous studies. The common indicators of lower socioeconomic status include lower level of education or lack of education, financial issue, and employment status, among others. In prior reports of CKD patients, lower socioeconomic status and lower educational levels were commonly associated with psychological problems [26,27]. In our study, we did not find significant differences in anxiety, depression, and sleep disturbance scores depending on educational level or the lack thereof in participants who had received unplanned HD. Similar findings were also noted in the analyses of marital status, religious status, and employment status. There were also inconsistent conclusions in the previous studies regarding the associations between psychological disturbance and gender and age in CKD patients [28,29]. Racial and medical insurance disparity were also considered to influence the outcomes in HD patients. A lower setting in economic status could delay nephrologists’ referral in CKD patients especially in low developed countries [30]. As a consequence, a sudden HD event may spur an untoward psychological reaction. Similarly, various medical insurance models participated by different socioeconomic population also discloses diverse outcomes in HD patients [31,32]. In our study, we observed no significant differences in anxiety, depression, or sleep disturbances scores based on gender and age stratification. Plausible explanations for the inconsistent findings in the sociodemographic relationship in our study, compared to previous studies, include different racial populations, cultural backgrounds, study criteria, and small sample size. It is worth investigating this issue with a more detailed analysis of patients with unplanned HD in the future, in order to provide holistic patient care.

Commonly, it is reasonable to expect anxiety and depression could influence sleep quality in CKD patients. In the past years, a few studies have shown depression contributing to poor sleep quality in non-dialysis CKD patients [33–36]. In contrast, anxiety was not demonstrated significant association with sleep disturbance in non-dialysis CKD patients [36]. In this study, we found anxiety and depression exhibited significant correlation with sleep disturbance in unplanned HD patients (Supplementary Table 1). We hypothesized that a sudden event for unplanned HD could result in mutual interaction by aforementioned factors. Therefore, a poor sleep quality may develop in unplanned HD patients. We expect more studies in the future to define these associations in unplanned HD patients.

Patients with CKD often suffer from many comorbidities. This multi-morbid status indeed influences the psychological reaction of CKD patients. Deteriorated organ function leads to disability in daily activities, performance status, and unsatisfactory emotional reaction [37,38]. The most common disease entities impacting psychological disturbance in the unplanned dialysis setting include diabetes, coronary artery disease, and congestive heart failure [12]. In our study, we could not find that the number of comorbidities significantly influenced anxiety, depression, and sleep disturbance scores. Hypertension was the only comorbidity that influenced the anxiety score by generalized linear regression analysis. However, we did not include disease severity and vintage, and concomitant medication in these comorbid diseases. Therefore, the specific impact of psychological disturbance could not be determined in our participants. A sophisticated scoring system for disease evaluation can be expected in the future to investigate their impact on psychological burden in patients undergoing unplanned dialyses.

The association of anxiety, depression, and sleep disturbance with various laboratory parameters was examined in our study. Overall, laboratory parameters were not associated with psychological distress. It is reasonable to assume psychological distress from disease burden, not from levels of laboratory parameters. We can conclude from our findings that therapeutic strategies to alleviate disease burden and fear of dialysis therapy are the main instruments for lessening psychological distress in this CKD population.

There is evidence that the clinical benefits of a multidisciplinary CKD program include postponement of dialysis initiation [39,40]. However, unplanned dialysis initiation is still common among advanced renal failure patients, even with follow-up in a multidisciplinary CKD program [12]. We found a significant association between a multidisciplinary CKD care program in outpatient clinic with anxiety, depression, and sleep disturbance in unplanned HD patients in our study. We speculated that a continuous psychological burden could result in unexpected timing for renal replacement therapy. Accordingly, timely education and dialysis access for CKD patients may reduce the severity of psychological shock in patients with advanced CKD.

The limitations of the study include lack of a placebo arm, psychological evaluation only at one time-point, and relatively short follow-up. Additionally, evaluation by self-rating scales may capture somatic symptoms that are not necessarily indicative of psychological problems and may, consequently, be overestimated in our cohort. Finally, our study was limited to one tertiary hospital. Thus, our experience may not be extrapolatable to other patient populations and different practice patterns. The strengths of this study include prospective systematic screening for anxiety, depression, and sleep disturbance in patients receiving unplanned HD, data entry with continuous verification of the accuracy, and good adherence to the study protocol with little loss to follow-up.

## Conclusions

Our study showed a heavy psychological burden in unplanned HD patients indicated by anxiety, depression, and sleep disturbance. These psychological disturbances did not exhibit a significant association with sociodemographic profile. A multidisciplinary CKD care program in outpatient clinic can reduce the severity of psychological disturbances when CKD patients undergo unplanned HD.

## Supplementary Material

Supplemental Material

## Data Availability

The raw data used to support the findings of this study are available from the corresponding author upon reasonable request.
