# Supplementary material for: Correlation of sociodemographic profiles with psychological problems among hospitalized patients receiving unplanned hemodialysis
Source: Ren Fail. 2020 Mar 9;42(1):255–62. doi: 10.1080/0886022X.2020.1736097 (PMC7144224; doi:10.1080/0886022X.2020.1736097)
Supplement: Supplemental Material [file IRNF_A_1736097_SM4704.pdf]

Supplemental Table 1. Correlation analysis among anxiety, depression, and sleep disturbance.

Variables	Depression scores		Sleep disturbance scores	
	r	*P*	*r*	*P*
Anxiety scores	.893	<.001	.751	<.001
Depression scores			.765	<.001

P-value estimated using Pearson's correlation test.

r : Pearson's correlation coefficient.
